# Designing accurate emulators for scientific processes using calibration-driven deep models

**DOI:** 10.1038/s41467-020-19448-8

**Published:** 2020-11-06

**Authors:** Jayaraman J. Thiagarajan, Bindya Venkatesh, Rushil Anirudh, Peer-Timo Bremer, Jim Gaffney, Gemma Anderson, Brian Spears

**Affiliations:** 1 Lawrence Livermore National Laboratory, Center for Applied Scientific Computing, Livermore, CA USA; 2 School of Electrical, Computer and Energy Engineering, Arizona State University, Tempe, AZ USA

**Keywords:** Computer science, Scientific data

## Abstract

Predictive models that accurately emulate complex scientific processes can achieve speed-ups over numerical simulators or experiments and at the same time provide surrogates for improving the subsequent analysis. Consequently, there is a recent surge in utilizing modern machine learning methods to build data-driven emulators. In this work, we study an often overlooked, yet important, problem of choosing loss functions while designing such emulators. Popular choices such as the mean squared error or the mean absolute error are based on a symmetric noise assumption and can be unsuitable for heterogeneous data or asymmetric noise distributions. We propose Learn-by-Calibrating, a novel deep learning approach based on interval calibration for designing emulators that can effectively recover the inherent noise structure without any explicit priors. Using a large suite of use-cases, we demonstrate the efficacy of our approach in providing high-quality emulators, when compared to widely-adopted loss function choices, even in small-data regimes.

## Abstract (web summary)

The success of machine learning for scientific discovery normally depends on how well the inherent assumptions match the problem in hand. Here, Thiagarajan et al. alleviate this constraint by allowing the change of optimization criterion in a data-driven approach to emulate complex scientific processes.

## Introduction

Building functional relationships between a collection of observed input variables **x** = {*x*_1_, ⋯ , *x*_*d*_} and a response variable **y** is a central problem in scientific applications—examples range from estimating the future state of a molecular dynamics simulation^1^ to searching for exotic particles in high-energy physics^2^ and detecting the likelihood of disease progression in a patient^3^. Emulating complex scientific processes using computationally efficient predictive models can achieve significant speedups over traditional numerical simulators or conducting actual experiments, and more importantly provides surrogates for improving the subsequent analysis steps such as inverse modeling, experiment design, etc. Commonly referred to as supervised learning in the machine- learning literature, the goal here is to infer the function *f*: **x** ↦ **y** using a training sample ${\{({{\bf{x}}}_{i},{{\bf{y}}}_{i})\}}_{i = 1}^{n}$, such that the expected discrepancy between **y** and *f*(**x**), typically measured using a loss function ${\mathcal{L}}({\bf{y}},f({\bf{x}}))$, is minimized over the joint distribution *p*(**x**, **y**).

With the availability of modern representation-learning methods that can handle complex, multivariate datatypes, the response variable **y** can now correspond to quantities ranging from a collection of scalars, to images, multivariate time-series measurements, and even symbolic expressions, or combinations thereof^4–7^. In particular, the success of deep neural networks (DNN) in approximating scientific processes involving different types of response variables has generated significant research interest toward improving the accuracy and reliability of emulators^8–11^. This includes the large body of recent works on incorporating known scientific priors as constraints into predictive modeling^12^, designing custom neural network architectures that can systematically preserve the underlying symmetries^13^, integrating uncertainty quantification methodologies to improve model reliability^9^, and devising novel learning techniques that can handle the inherent data challenges in scientific problems (e.g., small data, underdetermined systems)^8^. However, a fundamental, yet often overlooked, aspect of this problem is the choice of the loss function ${\mathcal{L}}$. Denoting **y** = *f*(**x**) + *n*, where *n* denotes the inherent noise in the observed data, the loss function used to measure the discrepancy **y** − *f*(**x**) is directly linked to the assumptions made on the noise distribution.

Despite the importance of ${\mathcal{L}}$ in determining the fidelity of *f*, in practice, simple metrics, such as the *ℓ*_2_-metric, ∣∣**y** − *f*(**x**)∣∣_2_, are used, mostly for convenience but also due to lack of priors on the distribution of residuals. However, this disregards the inherent characteristics of the training data and more importantly the fact that choosing a metric implicitly defines a prior for *n*. Yet, appropriately accounting for noise is crucial to robustly estimate *f* and to create high-fidelity predictions for unseen data. However, this assumption can be easily violated in real-world data. For example, the *ℓ*_2_ metric is known to be susceptible to outliers^14^ and cannot handle fast-state dynamics such as jumps in the state values^15^. A potential solution is to resort to other symmetric loss functions, e.g., Huber^14^ or the Vapnik’s *ϵ*—insensitive loss^16^, that are known to be more robust. However, even those variants can be insufficient when data are more heterogeneous, for example, due to heteroscedastic variance or other forms of non-location-scale covariate effects^17^. With heterogeneous data, merely estimating the conditional mean is insufficient, as estimates of the standard errors are often biased. This has led to the design of different parameterized, asymmetric loss functions, e.g., quantile^17^ or quantile Huber^18,19^, that enable one to explore the entire conditional distribution of the response variable *p*(**y**∣**x**) instead of only the conditional mean. Though quantile regression has been found to be effective in handling heterogeneous data and being robust to outliers, determining the appropriate quantile parameter that reflects the expected degree of asymmetry in the distribution of residuals is challenging. This becomes even more intractable when the response variable **y** is multivariate, and one needs to determine the parameter *τ* for each of the response variables.

In this paper, we present Learn-by-Calibrating (LbC), a nonparametric approach based on interval calibration for building emulators in scientific applications that are effective even with heterogeneous data and are robust to outliers. The notion of interval calibration comes from the uncertainty quantification literature^20,21^ and can be formally defined as follows: let us assume that the model *f* is designed to produce prediction intervals, in lieu of simple point estimates, for the response **y**, i.e., $[\hat{{\bf{y}}}-{\delta }^{l},\hat{{\bf{y}}}+{\delta }^{u}]$. Suppose that the likelihood for the true response **y** to be contained in the prediction interval is $p(\hat{{\bf{y}}}-{\delta }^{l}\le {\bf{y}}\le \hat{{\bf{y}}}+{\delta }^{u})$, the intervals are considered to be well-calibrated if the likelihood matches the expected confidence level. For a confidence level *α*, we expect the interval to contain the true response for 100 × *α*% of realizations from *p*(**x**). Though calibration has been conventionally used for evaluating and correcting uncertainty estimators, this paper advocates for utilizing calibration as a training objective in regression models. More specifically, LbC uses two separate modules, implemented as neural networks, to produce point estimates and intervals, respectively, for the response variable, and poses a bilevel optimization problem to solve for the parameters of both the networks. This eliminates the need to construct priors on the expected residual structure and makes it applicable to both homogeneous and heterogeneous data. Furthermore, by effectively recovering the inherent noise structure, LbC leads to highly robust models.

**Fig. 1:**
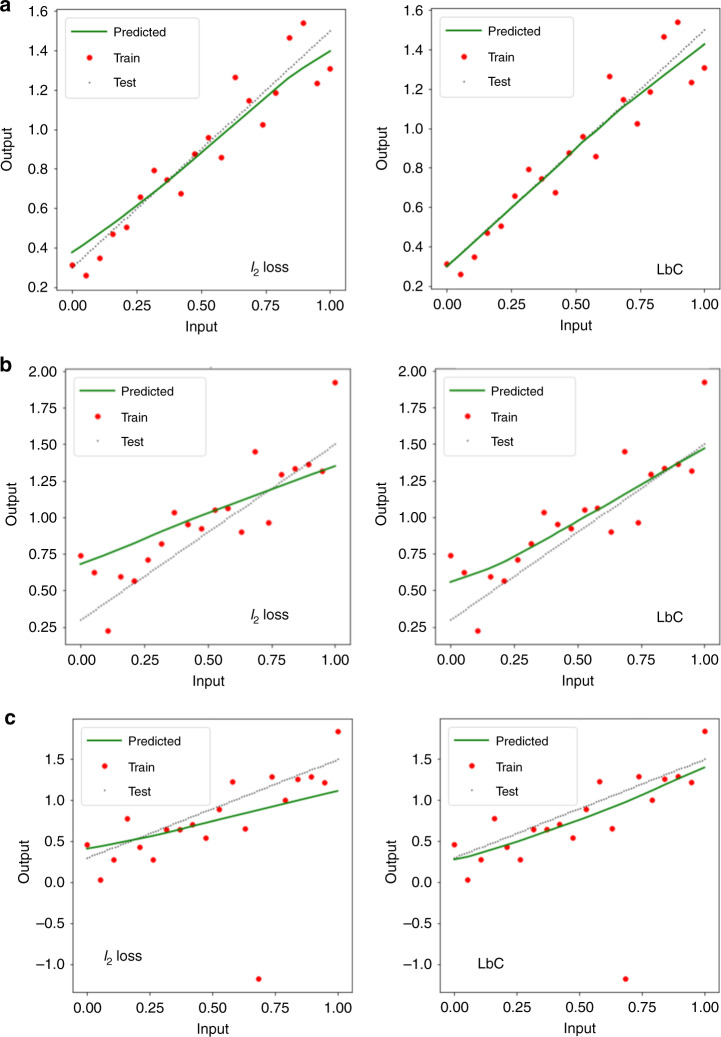
Illustration with a 1D regression example. Comparing models trained using the symmetric *ℓ*_2_ loss and Learn-by-Calibrating (LbC). **a** When the noise model for the observed data is symmetric (Gaussian in this case), even the standard MSE loss can recover the true function. **b** When the noise model is asymmetric (positive skew), symmetric losses lead to poor approximations. In contrast, LbC can produce higher-fidelity predictions by not enforcing a symmetric residual structure. **c** When there are outliers in addition to an asymmetric (negative skew) noise model, the nonrobustness of the squared error metric becomes clearly evident, while LbC is found to be robust.

  Figure 1 provides an illustration of a simple 1D regression experiment using a single-layer neural network with 100 neurons and rectified linear units (ReLU) nonlinear activation. We find that LbC is consistently superior to the widely adopted *ℓ*_2_ and Huber loss functions, under both symmetric and asymmetric noise models, as well as in the presence of outliers. Note that the evaluation metric in each of the examples (and throughout the paper) remains the traditional MSE and the R-squared (*R*^2^) statistic. The only difference is the loss function used during training. We attribute this improvement to the data-driven noise model of the LbC objective that generalizes better to unseen data.

We evaluated the proposed approach using a large suite of use cases, which require the design of accurate emulators for the underlying scientific processes. These benchmarks represent a broad range of real-world scenarios including different sample sizes, varying input dimensionality, and the need to handle response variable types ranging from single/multiple scalar quantities and multivariate time-series measurements to multimodal outputs. Our empirical studies clearly demonstrate the effectiveness of calibration-based training in inferring high-fidelity functional approximations to complex scientific processes. We find that it consistently outperforms several state-of-the-art baselines, including different variants of DNN and ensemble techniques, such as random forests and gradient-boosting machines, trained with the widely adopted MSE and Huber loss functions. Furthermore, when compared to deep networks trained with the symmetric losses, we find that LbC can operate reliably even in small-data regimes (as low as 1000), producing higher-quality models than even ensemble methods. In summary, LbC is a simple, yet powerful, approach to design emulators that are robust, reflect the inherent data characteristics, generalize well to unseen samples, and reliably replace accurate (expensive) simulators in scientific workflows.

## Results

The primary focus of this study is to investigate the impact of using a calibration-driven training objective, in lieu of widely adopted loss functions, on the quality of emulators. The problems that we consider encompass a broad range of applications, response types, and data sizes, and enable us to rigorously benchmark the proposed approach. Table 1 provides a description of datasets used in each of the use cases. For evaluation, we use two standard metrics, namely root mean-squared error (lower is better) and the R-squared statistic (*R*^2^), which measures the proportion of variance in the response variable that is predictable from the input variable (higher is better).

**Table 1:** Data description.

Test case	# Inputs	# Outputs	# Samples
Superconductivity	81	1	21,263
Airfoil self-noise	5	1	1503
Concrete	8	1	1030
Electric grid stability	12	1	10,000
Parkinsons	16	1	5875
ICF JAG (scalars)	5	15	10,000
ICF Hydra (scalars)	9	28	92,965
ICF Hydra (multi)	9	32	92,965
Reservoir model	14	14	2000

Use cases considered in our study for benchmarking the proposed approach.

### Data description

We consider a large suite of scientific problems and design emulators using state-of-the-art predictive modeling techniques, namely predicting the critical temperature of a superconductor based on its chemical formula^22^, airfoil self-noise estimation in aeronautical systems^23^, estimating compressive strength of concrete based on its material composition^24^, approximating a decentralized smart grid control simulation that characterizes the stability of an energy grid^25^, mimicking the clinical scoring process from biomedical measurements in Parkinson patients^26^, emulating a semi-analytical 1D simulator (JAG) for inertial confinement fusion that produces multiple diagnostic scalars^27^, emulating a 2D simulator for inertial confinement fusion that produces multimodal outputs, and emulating a reservoir simulator that provides estimates for oil-and-water production over time^28^.

Superconducting materials, which conduct current with zero resistance, are an integral part of magnetic resonance imaging (MRI) systems and utilized for designing coils to maintain high magnetic fields in particle accelerators. A superconductor exhibits its inherent zero-resistance property only at or below its critical temperature (*T*_c_). Developing scientific theory or a model to predict *T*_c_ has been an open problem, since its discovery in 1911, and hence empirical rules are used in practice. For example, it has been assumed that the number of available valence electrons per atom is related to *T*_c_, though there is recent evidence that this rule can be violated^29^. Hence, building statistical predictive models, based on a superconductor’s chemical formula, has become an effective alternative^22^. This dataset relates 81 elemental properties of each superconductor to the critical temperature on a total of 21,263 samples.

Controlling the noise generated by an aircraft, in particular the self-noise of the airfoil itself, is essential to improving its efficiency. The self-noise corresponds to the noise generated when the airfoil passes through smooth nonturbulent inflow conditions. The so-called Brooks model, a semiempirical approach for self-noise estimation, has been routinely used over 3 decades, though it is known to underpredict the noise level in practice. In recent years, data-driven models are being used instead^23^, and it is crucial to improve the fidelity of such an emulator. This dataset consists of 1503 cases and 5 features, including the frequency, angle of attack, and chord length to predict self-noise.

The key objective of the popular UCI benchmark Concrete is to estimate the compressive strength of concrete, which is known to be a highly nonlinear function of its age and material composition. Similar to many other problems in engineering, machine-learning approaches have been found to be superior to heuristic models for estimating the target function^24^. This falls under the class of small-data problems, by containing only 1030 samples in 8 dimensions representing the material composition, e.g., amount of cement and fly ash etc.

The Decentralized Smart Grid Control (DSGC) system is a recently developed approach for modeling changes in electricity consumption in response to electricity-price changes. A key challenge in this context is to predict the stability, i.e., whether the behavior of participants in response to price changes can destabilize the grid. This dataset contains 10,000 instances representing local stability analysis of the 4-node star system, where each instance is described using 12 different features^25^.

Parkinsons is the second most common neurodegenerative disorder after Alzheimers. Though medical intervention can control its progression and alleviate some of the symptoms, there is no available cure. Consequently, early diagnosis has become a critical step toward improving the patient’s quality of life^26^. With the advent of noninvasive monitoring systems in healthcare, their use for early diagnosis in Parkinson patients has gained significant interest. The goal of this use case is to predict the severity of disease progression, quantified via the Unified Parkinsons Disease Rating Scale (UPDRS), from speech signals (vowel phonotations). The dataset comprises 5875 patients represented using 16 different speech features.

ICF JAG^27^ is a semianalytical 1D simulator for inertial confinement fusion (ICF), which models a high-fidelity mapping from the process inputs, e.g., target and laser settings, to process outputs, such as the ICF-implosion neutron yield. The physics of ICF is predicated on interactions between multiple strongly nonlinear physics mechanisms that have multivariate dependence on a large number of controllable parameters. Despite the complicated, nonlinear nature of this response, machine-learning methods such as deep learning have been shown to produce high-quality emulators^8^. This dataset contains 10,000 samples with 5 input parameters and 15 scalar quantities in the response.

ICF Hydra is a 2D physics code used to simulate capsule-implosion experiments^30^. This has the physics required to simulate National Ignition Facility (NIF0 capsules, including hydrodynamics, radiation transport, heat conduction, fusion reactions, equations of state, and opacities). It consists of over a million lines of code and takes hours to run a single simulation. In terms of sample size, this is a fairly large-scale data with about 93 K simulations, where each sample corresponds to nine input parameters and a multimodal response (2-channel X-ray images, 28 scalar quantities, FNADS). In our experiments, we consider two different variants, one with only the multivariate scalar response and another with the entire multimodal response. Following the protocol in ref. ^8^, in the case of multimodal responses, we first build an encoder–decoder-style neural network that transforms the multimodal response into a joint latent space of 32 dimensions and repose the surrogate-modeling problem as predicting from the input parameters into the low-dimensional latent space. We can recover the actual response using the decoder model on the predicted latent representations.

The reservoir simulator that we used models a two-well waterflood in a reservoir containing two stacked-channel complexes. The model represents a deep-water-slope channel system, in which sediment is deposited in channel complexes as a river empties into a deep basin. A high-quality surrogate is required to solve the crucial task of history matching, an ill-posed inverse problem for calibrating model parameters to real-world measurements. The dataset contains 2000 simulations with 14 input parameters and 3 time histories corresponding to injection pressure, oil-, and water- production rates. Similar to the ICF Hydra case, we use an autoencoder model to transform the multivariate time-series response into a 14-dimensional latent space. Note that we use the network architecture in ref. ^31^ for designing the autoencoder.

### Performance evaluation

To provide statistically meaningful results, we performed fivefold cross-validation, carried out under three different random seeds (to create train-test splits for cross-validation), for each of the use cases, and report the average performance (along with standard deviations). For our empirical analysis, we consider the following baseline methods: Random forests (RF) with 100 decision trees trained using the *ℓ*_2_ metric; Gradient-boosting machines with 100 decision trees, trained using the *ℓ*_2_ loss function; DNN with 5 fully connected layers; a final prediction layer with dimensions corresponding to the response variable (details can be found in the Methods section). Note that we used the ReLU nonlinear activation after every hidden layer and optimized for minimizing the *ℓ*_2_ metric; a variant of the DNN model, referred as DNN (drp), wherein we introduce dropout-based epistemic uncertainty estimation during training (details can be found in the Methods section).

**Fig. 2:**
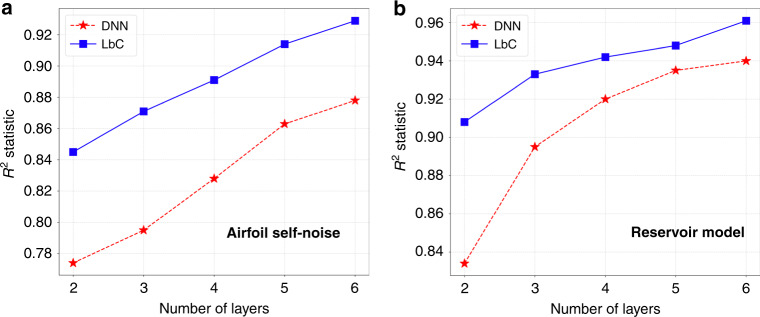
Impact of loss-function choice on model complexity. Comparing the performance of emulators designed using conventional deep neural networks (DNN) with MSE as the optimization objective and the proposed approach that utilizes a calibration objective: **a** airfoil self-noise dataset, **b** reservoir model dataset. We find that regardless of the complexity of the model (varying depth), the proposed approach produces improved emulators. Though Learn-by-Calibrating (LbC) uses an additional network for estimating the intervals during training, at inference time, the predictions are obtained using only the network *f* whose number of parameters are exactly the same as that of the DNN baseline.

**Fig. 3:**
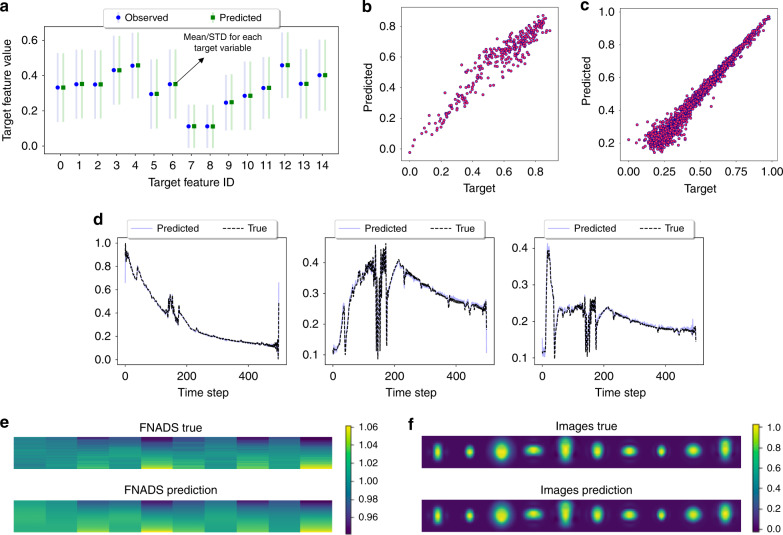
Qualitative evaluation of Learn-by-Calibrating (LbC) predictions. Predictions obtained using the proposed approach on different use cases: **a** ICF JAG—we show the distribution of values for each of the target variables and the corresponding predictions. **b** Airfoil self-noise. **c** Electric grid. **d** Reservoir model—reconstructions from the decoder. **e**–**f** FNADS and image predictions from the decoder for ICF Hydra. Across benchmarks of varying dimensionality and complexity, LbC produces high-fidelity emulators that can be reliably used in scientific workflows.

The RMSE and *R*^2^ scores achieved using the different approaches are reported in Tables 2 and 3, respectively. We find that LbC consistently produces higher-quality emulators in all cases, and comparatively lesser variance across different trials. In terms of the *R*^2^ statistic, we find that LbC achieves an average improvement of  ~8% over the popular ensemble methods, namely random forests and gradient-boosting machines, trained using the *ℓ*_2_ loss. On the other hand, when compared to the two deep-learning baselines, the average improvement in *R*^2^ is about 4%. Interestingly, with challenging benchmarks such as the Superconductivity and Parkinsons datasets, the standard neural network-based solutions (DNN, DNN (drp)) do not provide any benefits over conventional ensemble methods. This can be attributed to the overfitting behavior of overparameterized neural networks in small-data scenarios. In contrast, LbC is highly robust even in those scenarios and produces higher *R*^2^ scores (or lower RMSE). This is also apparent from the analysis in Fig. 2, where we find that even with a reduced number of parameters (number of layers), the proposed calibration-driven learning outperforms a standard deep model with 6 layers. This clearly emphasizes the discrepancy between the true data characteristics and the assumptions placed by the *ℓ*_2_ loss function. With simulators such as ICF Hydra and the reservoir model, which maps to complex response types, our approach makes accurate predictions in the latent space (from the autoencoder) and when coupled with the decoder accurately matches the true responses (Fig. 3). Interestingly, we find that LbC produces well-calibrated prediction intervals, when compared to widely adopted uncertainty-estimation methods, including Monte–Carlo dropout^32^, concrete dropout^33^, Bayesian neural networks (BNN)^34^, and heteroscedastic neural networks (HNN)^35^. Details of this comparison can be found in Supplementary Note 4.

**Table 2:** Surrogate model performance evaluation using root mean-squared error.

Test case	Methods				
	RF	GBT	DNN	DNN (drp)	LbC
Grid stability	0.063 ± 0.002	0.075 ± 0.003	0.057 ± 0.002	0.048 ± 0.002	**0.021** **±** **0.003**
Concrete	0.074 ± 0.04	0.081 ± 0.03	0.065 ± 0.016	0.065 ± 0.011	**0.046** **±** **0.008**
Parkinsons	0.068 ± 0.03	0.071 ± 0.04	0.063 ± 0.03	0.06 ± 0.04	**0.049** **±** **0.03**
Superconductivity	0.053 ± 0.02	0.064 ± 0.03	0.057 ± 0.02	0.048 ± 0.02	**0.039** **±** **0.02**
Airfoil self-noise	0.052 ± 0.018	0.069 ± 0.021	0.046 ± 0.015	0.041 ± 0.013	**0.031** **±** **0.011**
ICF JAG (scalars)	**0.007** **±** **4E-04**	0.009 ± 8E-04	0.01 ± 1E-03	0.008 ± 5E-04	**0.007** **±** **3E-04**
ICF Hydra (scalars)	0.012 ± 4E-03	0.016 ± 8E-03	0.011 ± 5E-03	0.01 ± 3E-03	**0.008** **±** **2E-03**
ICF Hydra (multi)	0.045 ± 5E-03	0.08 ± 9E-03	0.032 ± 4E-03	0.028 ± 3E-03	**0.019** **±** **3E-03**
Reservoir model	0.06 ± 6E-03	0.06 ± 7E-03	0.042 ± 2E-03	0.038 ± 2E-03	**0.029** **±** **3E-03**

The results were obtained over fivefold cross-validation, carried out using three different random seeds, on each of the use cases using emulators designed with different approaches. We report the mean and standard deviation across different trials, and the best performance in each case is denoted in bold.

**Table 3:** Surrogate model performance evaluation using *R*-squared statistic.

Test case	Methods				
	RF	GBT	DNN	DNN (drp)	LbC
Grid stability	0.89 ± 0.008	0.85 ± 0.007	0.94 ± 0.006	0.96 ± 0.003	**0.97** **±** **0.002**
Concrete	0.84 ± 0.22	0.82 ± 0.21	0.88 ± 0.13	0.89 ± 0.14	**0.91** **±** **0.09**
Parkinsons	0.71 ± 0.12	0.69 ± 0.14	0.7 ± 0.11	0.71 ± 0.13	**0.75** **±** **0.11**
Superconductivity	0.84 ± 0.17	0.79 ± 0.15	0.84 ± 0.19	0.86 ± 0.21	**0.89** **±** **0.13**
Airfoil self-noise	0.89 ± 0.11	0.81 ± 0.19	0.88 ± 0.12	0.9 ± 0.11	**0.94** **±** **0.06**
ICF JAG (scalars)	**0.995** **±** **0.002**	0.983 ± 0.003	0.975 ± 0.005	0.991 ± 0.002	**0.998** **±** **0.001**
ICF Hydra (scalars)	0.88 ± 0.015	0.81 ± 0.019	0.88 ± 0.08	0.89 ± 0.09	**0.94** **±** **0.08**
ICF Hydra (multi)	0.87 ± 0.011	0.81 ± 0.03	0.91 ± 0.01	0.95 ± 0.006	**0.97** **±** **0.008**
Reservoir	0.89 ± 0.004	0.87 ± 0.008	0.91 ± 0.01	0.93 ± 0.005	**0.96** **±** **0.006**

The results were obtained over fivefold cross-validation, carried out using three different random seeds, on each of the use cases using emulators designed with different machine-learning approaches. We report the mean and standard deviation across different trials, and the best performance in each case is denoted in bold.

**Fig. 4:**
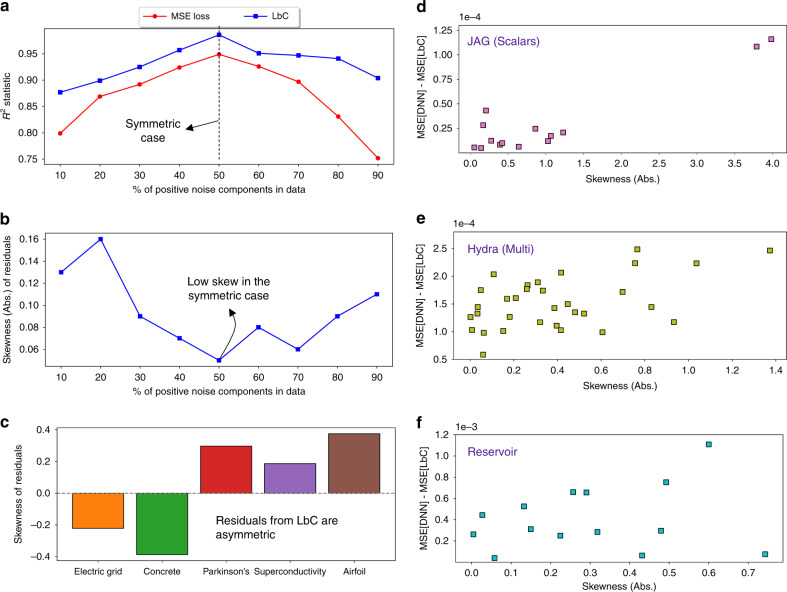
In-depth analysis of Learn-by-Calibrating (LbC). Using the synthetic function in Fig. 1, we find that **a** LbC produces significantly improved generalization at varying levels of asymmetry in the inherent noise structure and **b** the skewness of the residuals from LbC reflects that. For all the test cases considered in our study, **c** we find that the residuals are highly asymmetric and heavy-tailed. Interestingly, from figures **d**–**f**, we observe that, in cases where the performance gains are significant (difference between MSEs of deep neural networks (DNN) and LbC), the corresponding skewness of the residual distribution is high. This clearly evidences the ability of our approach to reveal the inherent noise structure in the data.

In contrast to existing loss functions, LbC does not place any explicit priors on the residual structure, and hence it is important to analyze the characteristics of errors obtained using our approach. Using the synthetic function from Fig. 1, we varied the percentage of positive noise components in the observed data (50% corresponds to the symmetric noise case) and evaluated the prediction performance using the *R*^2^ statistic. As shown in Fig. 4a, while LbC outperforms the MSE loss in all cases, with increasing levels of asymmetry, the latter approach produces significantly lower-quality predictions. This clearly evidences the limitation of using a simple Gaussian assumption or even a more general symmetric noise assumption, when the inherent noise distribution is actually asymmetric. From Fig. 4b, where we plot the skewness of residual distributions, we find that LbC effectively captures the true noise model, thus producing high-fidelity predictors. Furthermore, we make similar observations on the different use cases (see Fig. 4d–f)—the maximal performance gains (measured as the difference in MSE between the DNN baseline and LbC models with the same network architecture) are obtained when the skewness of the residuals from LbC is large, indicating the insufficiency of MSE loss in modeling real-world scientific data.

## Discussion

The intricate interactions between data sampling, model selection, and the inherent randomness in complex systems strongly emphasize the need for a rigorous characterization of ML algorithms^36,37^. In conventional statistics, uncertainty quantification (UQ) provides this characterization by measuring how accurately a model reflects the physical reality, and by studying the impact of different error sources on the prediction^35,38,39^. Consequently, several recent efforts have proposed to utilize prediction uncertainties in deep models to shed light onto when and how much to trust the predictions^35,40–43^. These uncertainty estimates can also be used for enabling safe ML practice, e.g., identifying out-of-distribution samples, detecting anomalies/outliers, delegating high-risk predictions to experts, and defending against adversarial attacks etc.

In recent years, a variety of estimators have been proposed in the literature for measuring these uncertainties in DNN, most often with classification models. For example, Bayesian neural nets^34^, Monte–Carlo dropout^32^, concrete dropout^33^, and ensembling techniques^44^ are commonly utilized to estimate the epistemic uncertainty (or model uncertainty). Similarly, Tagasovska et al. recently developed a conditional quantile-based estimator for measuring aleatoric uncertainties^45^. Due to the lack of suitable evaluation mechanisms for validating the quality of these estimates, it is common to utilize empirical calibration as a quality metric^20,46–49^. Interestingly, it has been reported in several studies that these estimators are not inherently well-calibrated^47^. Consequently, a large class of techniques that are aimed at calibrating pretrained models has been developed^50–53^. While these methods can produce well-calibrated prediction intervals in regression tasks, the estimated uncertainties cannot be directly utilized to update the model parameters. In contrast, this work proposes to utilize interval calibration to learn the model parameters and does not require a separate recalibration step. Using empirical studies with a number of benchmark problems in science and engineering, we find that LbC produces predictive models that are both accurate and well-calibrated (see Supplementary Note 4), when compared to existing uncertainty-estimation methods.

## Methods

### Formulation

LbC is a prior-free approach for training regression models via interval calibration. We begin by assuming that our model produces prediction intervals instead of simple point estimates, i.e., $[\hat{{\bf{y}}}-{\delta }^{l},\hat{{\bf{y}}}+{\delta }^{u}]$, for an input sample **x**. More specifically, our model comprises two modules *f* and *g*, implemented as DNN, to produce estimates $\hat{{\bf{y}}}=f({\bf{x}};\theta )$ and (*δ*^*l*^, *δ*^*u*^) = *g*(**x**; *ϕ*). We design a bilevel optimization strategy to infer *θ* and *ϕ*, i.e., parameters of the two modules, using observed data ${\{({{\bf{x}}}_{i},{{\bf{y}}}_{i})\}}_{i = 1}^{n}$:

1 $$\begin{array}{l}\mathop{\min }\limits_{\theta }{{\mathcal{L}}}_{f}\left(\theta ;{\{({{\bf{x}}}_{i},{{\bf{y}}}_{i})\}}_{i = 1}^{n},g({\phi }^{* })\right)\\ {\rm{s}}.{\rm{t}}.{\phi }^{* }=\arg \mathop{\min }\limits_{\phi }{{\mathcal{L}}}_{g}\left(\phi ;{\{({{\bf{x}}}_{i},{{\bf{y}}}_{i})\}}_{i = 1}^{n},f(\theta )\right).\end{array}$$

Here ${{\mathcal{L}}}_{f}$ and ${{\mathcal{L}}}_{g}$ are the loss functions for the two modules. In practice, we use an alternating optimization strategy to infer the parameters. LbC utilizes interval calibration from uncertainty quantification to carry out this optimization without placing an explicit prior on the residuals. We attempt to produce prediction intervals that can be calibrated to different confidence levels *α* and hence the module *g* needs to estimate (*δ*^*l*,*α*^, *δ*^*u*,*α*^) corresponding to each *α*. In our formulation, we use $\alpha \in {\mathcal{A}}$, ${\mathcal{A}}=[0.1,0.3,0.5,0.7,0.9,0.99]$. Note that while the choice of ${\mathcal{A}}$ is not very sensitive, we find that simultaneously optimizing for confidence levels in the entire range of [0, 1] is beneficial. However, considering more fine-grained sampling of *α*’s (e.g., {0.05, 0.1, ⋯ }) did not lead to significant performance gains, but required more training iterations. The loss function ${{\mathcal{L}}}_{g}$ is designed using an empirical calibration metric similar to^20^

2 $${{\mathcal{L}}}_{g}=	 \, \sum_{\alpha \in {\mathcal{A}}}\left(\left|\alpha -\frac{1}{n}\mathop{\sum }\limits_{i = 1}^{n}{\mathbb{1}}[{\hat{{\bf{y}}}}_{i}-{\delta }_{i}^{l,\alpha }\le {{\bf{y}}}_{i}\le {\hat{{\bf{y}}}}_{i}+{\delta }_{i}^{u,\alpha }]\right|\right.\\ 	 + \left.{\lambda }_{1}| {{\bf{y}}}_{i}-({\hat{{\bf{y}}}}_{i}-{\delta }_{i}^{l,\alpha })| +{\lambda }_{2}| ({\hat{{\bf{y}}}}_{i}+{\delta }_{i}^{u,\alpha })-{{\bf{y}}}_{i}| \right).$$

Here, $({\delta }_{i}^{l,\alpha },{\delta }_{i}^{u,\alpha })$ represents the estimated interval for sample index *i* at confidence level *α*, 1 is an indicator function, and *λ*_1_, *λ*_2_ are hyperparameters (set to 0.05 in our experiments). The first term measures the discrepancy between the expected confidence level and the likelihood of the true response falling in the estimated interval. Note that the estimates $\hat{{\bf{y}}}=f({\bf{x}};\theta )$ are obtained using the current state of the parameter *θ*, and the last two terms are used as regularizers to penalize larger intervals so that trivial solutions are avoided. In practice, we find that such a simultaneous optimization for different $\alpha ^{\prime}$s is challenging and hence we randomly choose a single *α* from ${\mathcal{A}}$ in each iteration, based on which the loss ${{\mathcal{L}}}_{g}$ is computed.

Since LbC relies entirely on calibration, there is no need for explicit discrepancy metrics like *ℓ*_2_ or Huber for updating the model *f*. Instead, we employ a hinge-loss objective that attempts to adjust the estimate $\hat{{\bf{y}}}$ such that the observed likelihood of the true response to be contained in the interval increases:

3 $${{\mathcal{L}}}_{f}=\mathop{\sum }\limits_{i = 1}^{n}{w}_{i}\left[\max (0,({\hat{{\bf{y}}}}_{i}-{\delta }_{i}^{l,\alpha })-{{\bf{y}}}_{i}+\gamma )+\max (0,{{\bf{y}}}_{i}-({\hat{{\bf{y}}}}_{i}+{\delta }_{i}^{u,\alpha })+\gamma )\right].$$

**Fig. 5:**
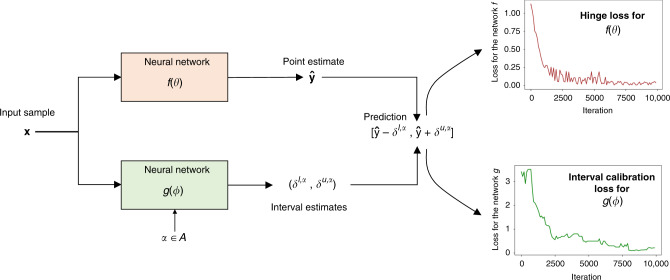
Learn-by-Calibrating (LbC) architecture overview. LbC uses are two separate networks to obtain point estimates and the intervals, respectively. As shown by the convergence plots during training, the two models synergistically optimize for the overall objective of improving the interval calibration.

Here, $({\delta }_{i}^{l,\alpha },{\delta }_{i}^{u,\alpha })=g({{\bf{x}}}_{i};\phi ,\alpha )$ is obtained using the recent state of the parameter *ϕ* and the randomly chosen *α* in the current iteration, *γ* is a predefined threshold (set to 0.05), and the weights ${w}_{i}=({\delta }_{i}^{l,\alpha }+{\delta }_{i}^{u,\alpha })/{\sum }_{j}({\delta }_{j}^{l,\alpha }+{\delta }_{j}^{u,\alpha })$ penalize samples with larger intervals. When compared to a competitive optimization algorithm, e.g., adversarial learning, in LbC, both models are working toward the common objective of improving interval calibration. In general, one can improve the calibration of predictions by adjusting the mean estimate to move closer to the true target, or by suitably widening the interval to include the true target even when the mean estimate is bad. Consequently, when the predictor model improves the mean estimates (for a fixed-interval estimator), the current interval estimates become overly optimistic, i.e., even at lower confidence levels, it will produce higher empirical confidence. Hence, in the subsequent iteration, the interval estimator will sharpen the intervals to make the estimates more underconfident, i.e., at higher confidence levels (say 0.9 or 0.99), it might provide lower empirical confidence. Consequently, LbC alternatively adjusts the predictor and interval estimator models to produce predictive models that are both accurate (good-quality mean estimates) and well-calibrated (at all confidence levels). This synergistic optimization process thus leads to superior quality predictions, which we find to be effective, regardless of the inherent residual structure. Figure 5 illustrates the proposed approach and the convergence curves for the two models *f* and *g* obtained for the synthetic example in Fig. 1.

### Architecture

In our implementation, both *f* and *g* are implemented as neural networks with fully connected layers and ReLU nonlinear activation. For use cases with at least 5000 samples, we used 5 fully connected layers and the number of hidden units fixed at [64, 128, 512, 256, 32], respectively, and a final prediction layer. Whereas, we used shallow 3-layer networks for the smaller datasets ([64, 256, 32]). While the final layer in *f* corresponds to the dimensionality of the response variable, the final layer in *g* produces *δ*^*l*^ and *δ*^*u*^ estimates for each dimension in **y** at every $\alpha \in {\mathcal{A}}$.

### Training

The networks were trained using the Adam optimizer with the learning rates for the two modules fixed at 1e − 5 and 1e − 4, respectively, and mini-batches of size 8. The alternating optimization was carried out for about 1000 iterations with a training schedule of (2,1), i.e., in each iteration, the predictor model is trained for two epochs, while the interval estimator is trained for one epoch. Though both models can be updated using the entire training dataset, in some cases, we find that improved test performance can be achieved by using separate data partitions. Similar ideas are used in meta-learning algorithms (e.g., MAML^54^) in order to implicitly measure the validation performance during training. In our experiments, we randomly split the data into two 50% partitions and used them for training the predictor and interval estimator models. Details of the hyperparameter choices and strategies for improved convergence of this alternating optimization are discussed in Supplementary Note 1.

### Baselines

Model ensembles constructed using random forests and gradient-boosting machines are known to be a strong baseline in regression problems^55^. Hence, we chose those two baselines to benchmark the performance of LbC. In addition, we considered standard DNN trained with the *ℓ*_2_ loss and a stae-of-the-art variant that incorporates Monte–Carlo dropout-^56^ based uncertainty estimation. Dropout is a popular regularization technique that randomly drops hidden units (along with their connections) in a neural network. Following^56^, for each sample, we make *T* forward passes with the dropout rate set to *τ* and obtain the final prediction as the average from the *T* runs. This is known to produce more robust estimates in regression problems^20^. In our experiments, we set *T* = 20 and the dropout rate *τ* = 0.3.

## Supplementary information

Supplementary Information

## Data Availability

All datasets used in this study, except for the ICF Hydra and reservoir model datasets, are publicly available and we have provided appropriate references to obtain them. The two proprietary datasets will be made available in the future.

## Code availability

The software codes associated with this paper will be hosted through a public code repository, https://github.com/jjayaram7/learn-by-calibrating.
