# Supplementary material for: Designing accurate emulators for scientific processes using calibration-driven deep models
Source: Nat Commun. 2020 Nov 6;11:5622. doi: 10.1038/s41467-020-19448-8 (PMC7648787; doi:10.1038/s41467-020-19448-8)
Supplement: Supplementary file 1 — Supplementary Information [file 41467_2020_19448_MOESM1_ESM.pdf]

Supplementary Material

Designing Accurate Emulators for Scientific Processes using Calibration-Driven Deep Models

Jayaraman J. Thiagarajan, Bindya Venkatesh, Rushil Anirudh, Peer-Timo Bremer, Jim Gaffney, Gemma Anderson, Brian Spears

Supplementary Note 1

We use an alternating optimization strategy to solve the bi-level objective of LbC. There are a few critical components that control the convergence behavior of this approach.

Choice of Training Schedule

The number of epochs that we run the update for each of the models is an important aspect to consider in alternating optimization strategies. Interestingly, we found that, a faster convergence can be achieved by having a non-uniform training schedule where the predictor model is updated twice more frequently than the interval estimator, i.e. in each iteration the predictor model is trained for 2 epochs while the interval estimator is trained for 1 epoch. On the other hand, updating the interval estimator more frequently (e.g. (1,2)) led to noisy training curves. This is particularly severe when the dataset sizes were small. In Supplementary Figure 1, we illustrate the effect of different training schedules. All results reported in the paper were obtained using the training schedule (2,1). Similar behavior was observed in all datasets considered in our study. Note, when the learning schedule was changed to $[3, 1]$, we did not observe any discernible difference when compared to a $[2, 1]$ schedule and we do not include that in the plot. However, changing the schedule to $[5, 1]$ made the convergence of the interval estimator significantly worse and hence the validation MSE was very poor.

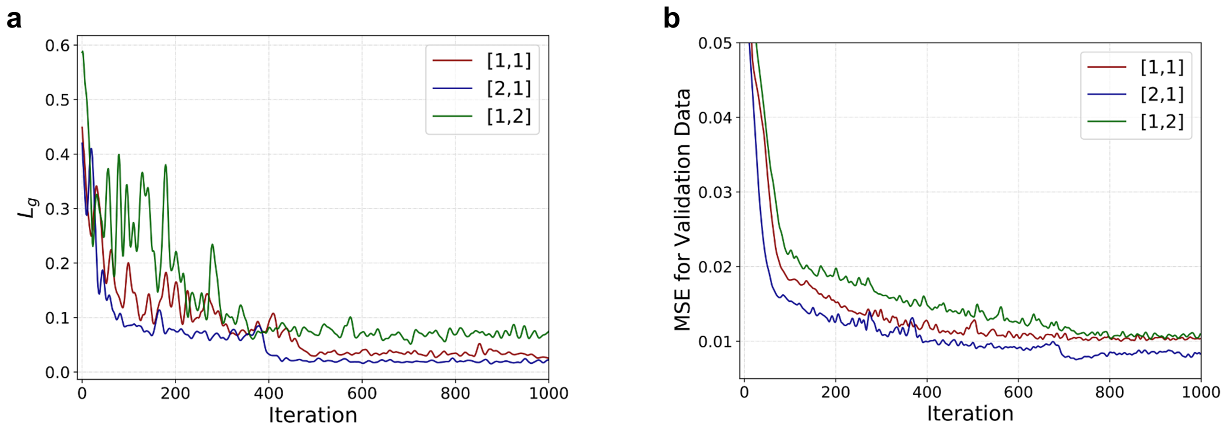

Supplementary Figure 1: Effect of the choice of learning schedule on the convergence of LbC: **a** We show the calibration error on the training data in different iterations for the Airfoil dataset; **b** We plot the MSE on the held-out validation set. We find that a learning schedule of [2,1], i.e., the predictor model being updated twice as frequently as the interval estimator, leads to improved convergence.

Choice of Batch Size

The batch size was found to be a crucial hyper-parameter for reliable convergence. We performed empirical studies to characterize its influence. More specifically, we varied the batch size between 8 and 128 and trained the models. As showed in Supplementary Figure 2, we find that, using larger batch sizes leads to overfitting behavior as demonstrated by rapid reduction in the training error, but a higher validation MSE. We also found that, using a smaller batch size led consistently better generalization on all datasets. However, reducing it further (batch size = 4) had a negative effect in at least some of the datasets, wherein the validation MSE plateaued at a higher value than what we obtained with batch size 8 or higher. All results reported in the paper were generated using a batch size of 8.

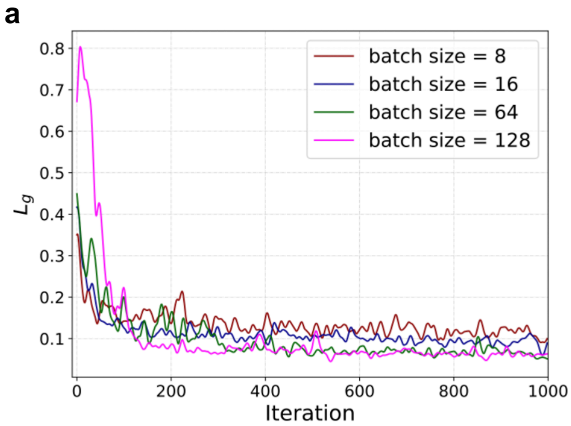

Supplementary Figure 2: Effect of the choice of batch size on the convergence of LbC. **a** We show the calibration error on the training data in different iterations for the Airfoil dataset at varying batch sizes; **b** we plot the MSE on the held-out validation set. We find that a lower batch size (8) provides better convergence on the validation set.

Choice of Confidence Levels for Optimization

For the calibration loss, we used the set of 6 confidence levels, $A = \{0.1, 0.3, 0.5, 0.7, 0.9, 0.99\}$. In general, we find that the simultaneous optimization of multiple confidence levels to be very challenging and hence in practice we resort to using a randomly chosen level in each iteration of LbC. Upon running the algorithm for a sufficient number of iterations, this strategy leads to prediction intervals that can calibrate to all confidence levels. However, with a more fine-grained choice of confidence levels, e.g. $A = \{0.05, 0.1, 0.2, 0.3, 0.4, 0.5, 0.6, 0.7, 0.8, 0.9, 0.95, 0.99\}$ (12 levels), the interval estimator model tends to produce noisy gradients (large variance) and requires a significantly larger number of iterations to converge. This is clearly apparent from the convergence plots in Supplementary Figure 3. Note that, in all cases, with sufficient number of iterations (2500 as opposed to 1000 iterations for the case of 6 levels), it still produces similar generalization performance.

Learning Rate Selection

Though the choice of learning rate for the interval estimator was not very sensitive, we found that using a lower learning rate for the predictor model produced stable convergence in all datasets. As illustrated in Supplementary Figure 4, using a lower learning for the predictor model ($lr_m = 1e-5, lr_d = 1e-4$) achieved a slower but stable convergence.

Effect of Data Partitioning

Though both models can be updated using the entire training dataset, in some cases, we find that improved test performance can be achieved by using separate data partitions. Similar ideas are used in meta-learning

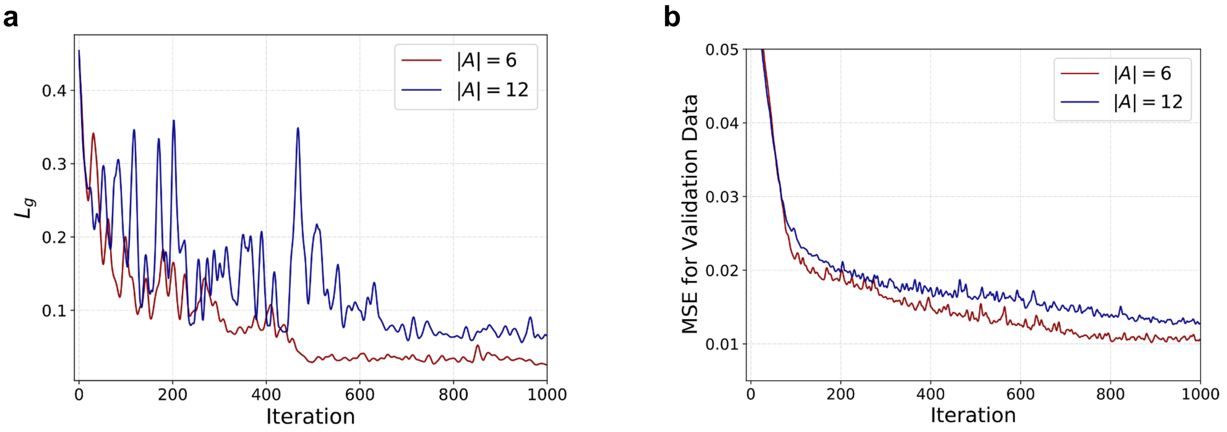

Supplementary Figure 3: Effect of the number of confidence levels (cardinality of the set A denoted by $|A|$) on the convergence of LbC. **a** We show the calibration error on the training data in different iterations for the Airfoil dataset at $|A| = 6$ and $|A| = 12$ respectively; **b** we plot the MSE on the held-out validation set. We find that using fine-grained confidence levels requires much larger number of iterations to converge.

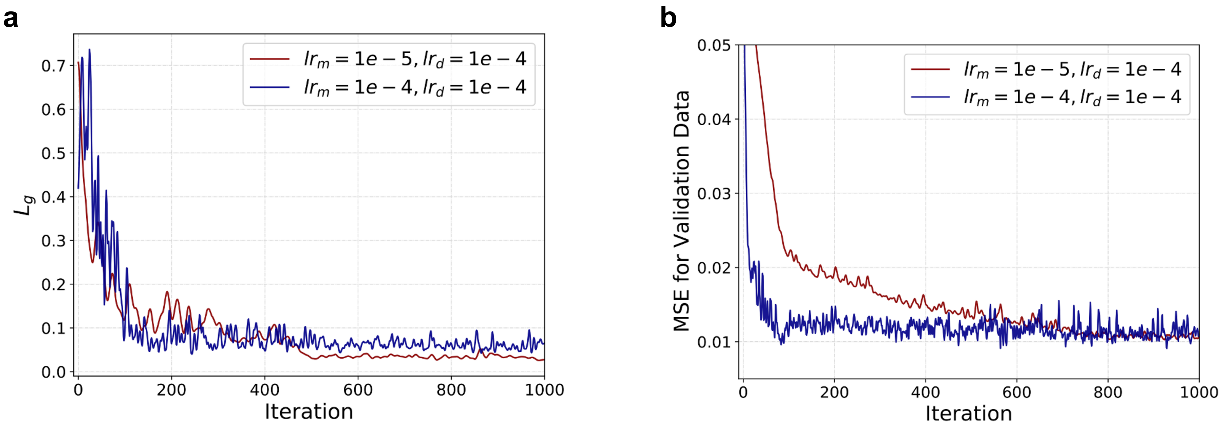

Supplementary Figure 4: Effect of the choice of learning rates for the two models on the convergence on LbC. We find that using a lower learning rate for the predictor model produced a more stable (but slower) convergence.

algorithms (e.g. MAML) in order to implicitly measure the validation performance during training. In our experiments, we randomly split the data into two 50% partitions and used them for training the predictor and interval estimator models. As showed in Supplementary Figure 5 for the Electric Grid Stability dataset, using data partitioning led to a much faster convergence. However, note that, in cases with small number of samples, we used the entire training data for both models.

Supplementary Note 2

The ℓ_2 loss is the arguably the most popular choice for building predictive models in scientific and engineering problems. However, it has been well-studied in the literature that the ℓ_2 loss is non-robust to outliers and can be appropriate when the noise (i.e., residual) in the data is asymmetric. This is illustrated using a synthetic example in Supplementary Figure 1 of the main paper, where we considered a linear function. To better illustrate the behavior of LbC in comparison to the ℓ_2 loss, we consider another $1D$ example with a

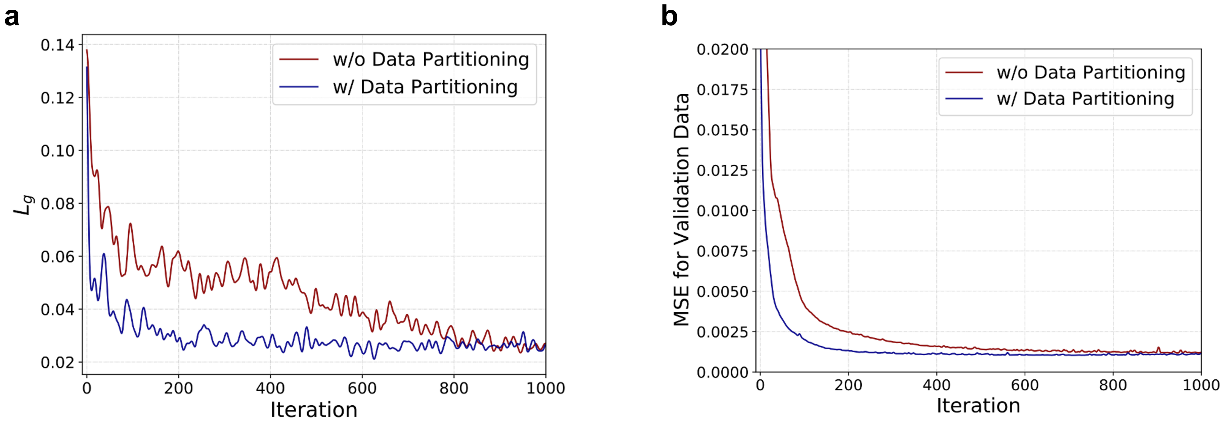

Supplementary Figure 5: Impact of data partitioning on the convergence of LbC: **a** We show the calibration error on the training data in different iterations for the Electric Grid Stability dataset with and without data partitioning; **b** we plot the MSE on the held-out validation set. We find that using different randomly chosen training data partitions for the two models leads to faster convergence.

non-linear function of the form:

$$y = \frac{1}{1 + \exp -a(x-b)} + c,$$

where $a = 0.2$, $b = -3.5$ and the offset $c = -0.35$. Here, we varied $x \in [0, 20]$. We considered the following 4 scenarios: (i) no noise added; (ii) Gaussian noise with $\sigma = 0.2$ added; (iii) No noise added but two outlying samples included; (iv) Asymmetric noise (skewed towards negative) added. In all cases, we used a fully connected network with 1 hidden layer of 100 neurons and ReLU activation. As expected, in the no noise and Gaussian noise cases, both ℓ_2 loss and LbC recover the underlying function accurately, wherein the former produces higher R^2 score. This clearly illustrates the regimes where ℓ_2 is a good choice. However, in the presence of outliers and asymmetric noise, we find that LbC is superior to ℓ_2. By making less strict assumptions on the residual structure, LbC produces improved performance on all real-word datasets considered.

Even with a simple dataset such as Concrete, where models such as decision trees or random forests are known to be effective, under certain train-test splits (based on the random seed used) ℓ_2 loss provides a very poor fit ($R^2 = 0$) (see Supplementary Figure 7**a**). Even in those cases, LbC produces models that are of significantly better quality ($R^2 = 0.48$).

Supplementary Note 3

The improved performance of LbC in small data regimes and even with less complex models is an important benefit. As showed in Supplementary Figure 2 of the main paper, in one random trial, a 2-layer network with the LbC objective matches the performance of a 6-layer network with the ℓ_2 loss. Though the number of layers required by LbC to match the performance of a standard deep model varies across datasets and across random seeds used for creating the train-test splits, we find this general observation to hold – "even using a lesser number of parameters, LbC can produce better model fits". To demonstrate this, we performed 3 random trials for different datasets, at varying levels of model complexity, and report the mean R^2 scores in Supplementary Table 1.

Supplementary Note 4

In recent years, several classes of uncertainty estimation techniques have been proposed [1, 2, 3, 4] and these method produce prediction intervals in lieu of simple point estimates. A typical approach to evaluate prediction intervals (PIs) is via their calibration [5]: A PI is well calibrated if the likelihood of true target

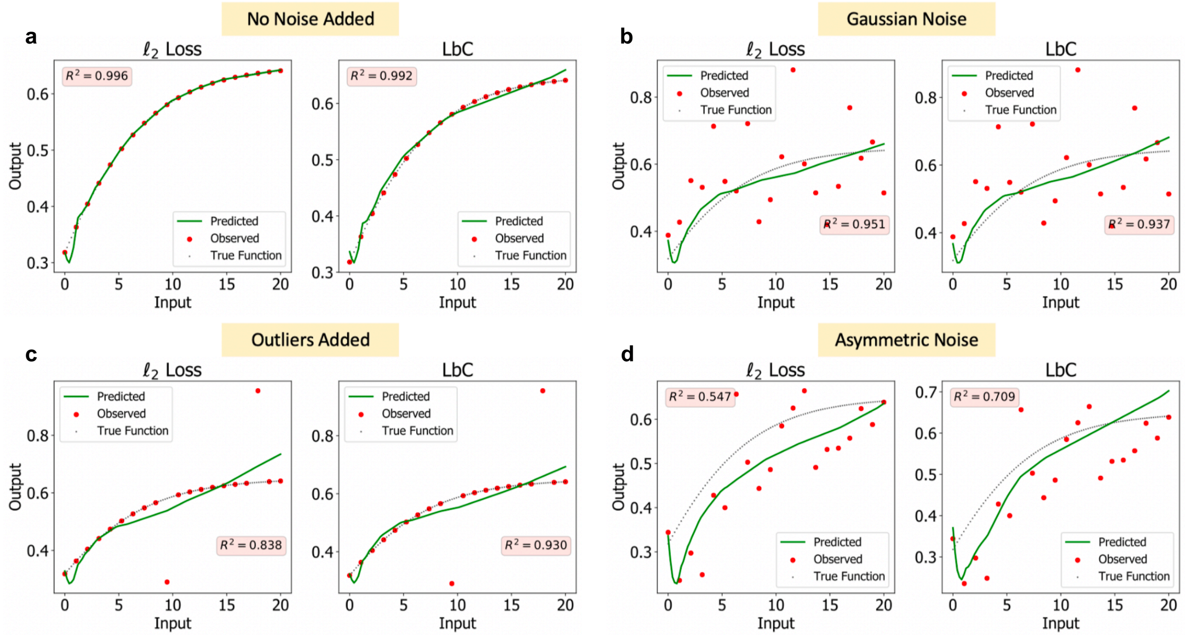

Supplementary Figure 6: Comparing LbC with ℓ_2 using a synthetic example with a non-linear function. While ℓ_2 is found to be highly effective when there is no noise in the data and the underlying noise process in Gaussian, LbC is consistently superior when there are outliers or asymmetric noise components in the data.

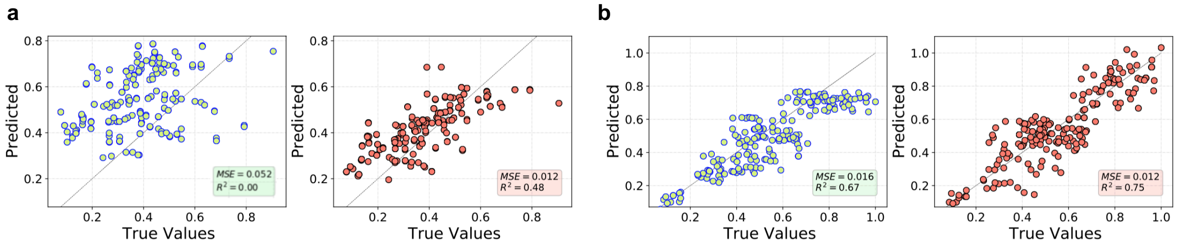

Supplementary Figure 7: **Comparing predictions from LbC with random forests**. **a-b** Predictions obtained using random forests (50 trees with an ℓ_2 loss) and LbC for two different random train-test splits of the concrete dataset. Even in cases (**a**) where a random forest with the ℓ_2 loss fails to recover the true function (due to poor data sampling), LbC is highly effective.

falling in the interval is consistent with the confidence level of the interval. In the context of calibration, most existing UQ methods in deep learning are known to be not inherently calibrated [6]. We measure the calibration error as follows:

$$ECE = \sum_{\alpha \in A} \left| \alpha - \frac{1}{N} \sum_{i=1}^{N} \mathbb{1} \left[(\hat{\mathbf{y}}_i - \delta_i^{l,\alpha}) \leq \mathbf{y}_i \leq (\hat{\mathbf{y}}_i + \delta_i^{u,\alpha}) \right] \right|, \tag{1}$$

We compare against the following baseline techniques that are commonly adopted to produce prediction intervals. Though there exists an entire class of recalibration methods [6], which utilizes an additional recalibration dataset to refine the learned estimator with a calibration objective, our focus is on single-shot calibration methods: MC Dropout [1]: In this approach, dropout in deep neural networks is cast as an approximate Bayesian inference in deep Gaussian processes. Using dropout during both train and test phases simulates Monte-Carlo sampling from the network with non-deterministic weights, thus capturing epistemic uncertainties. For all experiments, the dropout probability was set at 0.3; Concrete Dropout [2]: This was designed to automatically tune the dropout probability based on data characteristics, and hence

Dataset	2-Layers		4-Layers		6-Layers	
	DNN (drp)	LbC	DNN (drp)	LbC	DNN (drp)	LbC
Superconductivity	0.701	0.821	0.812	0.826	0.823	0.83
Airfoil Self-Noise	0.774	0.845	0.828	0.891	0.881	0.924
Concrete	0.715	0.883	0.877	0.892	0.889	0.895
Electric Grid Stability	0.864	0.958	0.956	0.969	0.967	0.971
Parkinsons	0.428	0.615	0.556	0.687	0.634	0.708
ICF JAG (Scalars)	0.971	0.989	0.986	0.994	0.992	0.994
ICF Hydra (Scalars)	0.846	0.909	0.897	0.927	0.903	0.942
ICF Hydra (Multi)	0.911	0.945	0.936	0.953	0.958	0.962
Reservoir Model	0.834	0.912	0.922	0.941	0.935	0.968

Supplementary Table 1: Comparison of standard deep models (trained with ℓ_2 loss) against LbC at varying levels of model complexity. We find that in all cases, a $4-$layer LbC model matches the performance of a $6-$layer standard deep regression model. Here, we show the mean R^2 scores from 3 random trials of $80 - 20$ train-test split.

it is expected to produce better calibrated intervals in comparison to MC Dropout; Heteroscedastic Neural Networks (HNN) [7]: Using the Gaussian likelihood formulation, the data dependent observation noise, i.e. aleatoric uncertainties, can be captured through the heteroscedastic loss; Bayesian Neural Networks (BNN) [8]: In this approach, the underlying distribution on network weights are approximated with a Gaussian (variational approximation), and the posterior inference based on observed data is then carried out by sampling from this approximate distribution, thus capturing epistemic uncertainties.

In Supplementary Table 2, we show the calibration error obtained using the different single-shot calibration strategies on all datasets considered in this study. Since LbC directly optimizes for the caliration objective, it achieves consistently lower calibration error compared to existing strategies.

Dataset	MC Dropout	Concrete Drp.	HNN	BNN	LbC
Superconductivity	0.49	0.57	0.61	0.51	**0.21**
Airfoil Self-Noise	0.32	0.27	0.39	0.19	**0.11**
Concrete	0.19	0.15	0.23	0.26	**0.07**
Electric Grid Stability	0.58	0.94	0.51	0.44	**0.36**
Parkinsons	1.09	1.22	0.89	0.77	**0.32**
ICF JAG (Scalars)	0.78	0.71	0.81	0.53	**0.17**
ICF Hydra (Scalars)	1.15	1.09	0.74	0.78	**0.29**
ICF Hydra (Multi)	1.44	1.23	0.91	0.76	**0.33**
Reservoir Model	0.98	1.19	0.58	0.47	**0.29**

Supplementary Table 2: Empirical Calibration Error (ECE) obtained using popular deep uncertainty estimation techniques in comparison to LbC.

Supplementary Note 5

Using a number of benchmarks in science and engineering, we showed (in the main paper) that LbC consistently produces more accurate and calibrated predictive models. However, in all problems considered, we used only fully-connected networks to construct the predictor. Even in the cases of ICF Hydra (Multi) and Reservoir Model with complex output types (images and time-series), we used pre-trained auto-encoding models to build multi-variate representations that can be fit using fully connected networks. However, LbC

is a generic learning approach that can be applied to any network architecture or type of data. In order to demonstrate this, we consider two different use-cases - (i) object detection in images using a convolutional neural network; (ii) time-series forecasting using an LSTM model.

Image Data

We pose the problem of object localization as predicting a bounding box around the object using a convolutional neural network. Each bounding box is characterized by 4 regression targets – (x, y) coordinates of the left-bottom corner, height h and width w. For this experiment, we used the Caltech Birds (CUB) dataset [9], which is comprised of $11,788$ images belonging 200 different categories of birds. We performed transfer learning from a ResNet-18 model, pre-trained on imagenet classification, to carry out this task. We allowed fine-tuning of the last residual block and the final prediction layer during training. As seen in Supplementary Table 3, LbC significantly outperforms standard CNN models trained with the ℓ_2 loss (and optionally Monte Carlo dropout) in terms of the IoU score (Intersection over Union between the true and estimated bounding boxes of the object).

**Method**	**IoU Score**
CNN + ℓ_2 loss	0.468
CNN (drp) + ℓ_2 loss	0.473
CNN + LbC	**0.561**

Supplementary Table 3: Object detection with convolutional neural networks (CNN). We build ResNet-18 models to predict bounding boxes around objects in an image. Posed as a regression task of estimating coordinates of the bounding boxes, a well-calibrated model (LbC) produces significantly improved estimates over standard neural networks (with ℓ_2 loss).

Time-Series Data

We consider the problem of forecasting in time-varying data by leveraging information from the past samples. More specifically, we attempt to look-ahead by one sample $\mathrm{x}[t+1]$ using the observations $\mathrm{x}[t-k:t+1]$. For this empirical study, we considered 4 datasets, namely NSW2013 and TAS2016 annual electricity demand recordings from the Australian Energy Market Operator, air quality and Beijing PM2.5 datasets from UCI [10]. In each case, we used $k = 24$ steps in the past to look ahead, and overall we used the first 30% of the time-steps for training and the rest for testing. Our architecture comprised of a RNN with LSTM units (2 layers with 128 hidden dimensions) and a linear layer for making the final prediction. As showed in Supplementary Table 4, LbC consistently produces higher-quality predictions in all cases (indicated by lower RMSE).

**Method**	**NSW2013**	**TAS2016**	**Beijing PM 2.5**	**Air Quality**
LSTM + ℓ_2 loss	129.7	21.93	23.31	0.68
LSTM (drp) + ℓ_2 loss	125.48	22.12	22.42	0.66
LSTM + LbC	**89.72**	**17.64**	**18.51**	**0.523**

Supplementary Table 4: Time-series forecasting with LSTM models. We use a $2-layer$ LSTM model to make future predictions for different univariate time-series datasets. Here, we show the RMSE obtained in each of the cases using LbC and standard ℓ_2 error based models. Through the use of interval calibration for learning the model parameters, LbC produces high-fidelity estimates.

Supplementary References

[1] Gal, Y. & Ghahramani, Z. Dropout as a bayesian approximation: Representing model uncertainty in deep learning. In *International Conference on Machine Learning*, 1050–1059 (2016).

[2] Gal, Y., Hron, J. & Kendall, A. Concrete dropout. In *Advances in Neural Information Processing Systems*, 3581–3590 (2017).

[3] Lakshminarayanan, B., Pritzel, A. & Blundell, C. Simple and scalable predictive uncertainty estimation using deep ensembles. In *Advances in Neural Information Processing Systems*, 6402–6413 (2017).

[4] Ghahramani, Z. Probabilistic machine learning and artificial intelligence. *Nature* **521**, 452 (2015).

[5] Heskes, T. Practical confidence and prediction intervals. In *Advances in Neural Information Processing Systems*, 176–182 (1997).

[6] Kuleshov, V., Fenner, N. & Ermon, S. Accurate uncertainties for deep learning using calibrated regression. *arXiv preprint arXiv:1807.00263* (2018).

[7] Kendall, A. & Gal, Y. What uncertainties do we need in bayesian deep learning for computer vision? In *Advances in Neural Information Processing Systems*, 5574–5584 (2017).

[8] Blundell, C., Cornebise, J., Kavukcuoglu, K. & Wierstra, D. Weight uncertainty in neural networks. In *Proceedings of the 32nd International Conference on Machine Learning*, 1613–1622 (2015).

[9] Welinder, P. *et al.* Caltech-UCSD Birds 200. Tech. Rep. CNS-TR-2010-001, California Institute of Technology (2010).

[10] Dua, D. & Graff, C. UCI machine learning repository (2017). URL `http://archive.ics.uci.edu/ml`.
